# AI-driven network pharmacology and multi-omics validation identify KCNH2 as a prognostic biomarker and candidate therapeutic vulnerability of Acorus tatarinowii in glioblastoma

**DOI:** 10.3389/fphar.2026.1811032

**Published:** 2026-04-23

**Authors:** Xiaoqing Song, Yuanyang Xiao, Junhui Liu, Shanshan Wei, Meijuan Tan, Yuanyuan Chen, Xiaohong Yu

**Affiliations:** 1 Department of Oncology, Wuchang Hospital Affiliated to Wuhan University of Science and Technology, Wuhan, China; 2 Vascular Surgery, Wuhan No. 9 Hospital, Wuhan, China; 3 Department of Neurosurgery, Renmin Hospital of Wuhan University, Wuhan, Hubei, China

**Keywords:** artificial intelligence, biomarker, biomarker discovery, drug target, glioblastoma, KCNH2, multi-omics, network pharmacology

## Abstract

**Background:**

Glioblastoma (GBM) remains a highly aggressive malignancy with limited effective therapeutic options. Integrating traditional medicine resources with artificial intelligence–based analytical strategies may accelerate the identification of novel biomarkers and drug targets.

**Methods:**

We applied a network pharmacology framework to screen bioactive compounds of Acorus tatarinowii and identify intersecting targets with GBM-related genes. LASSO Cox regression was performed in TCGA-GBM to construct a prognostic model. Multi-omics analyses, including transcriptomic validation, CPTAC proteomics, copy-number alteration profiling, mutation landscape characterization, immune infiltration assessment, and pharmacogenomic correlation analysis, were conducted to prioritize key targets. Functional experiments based on genetic perturbation of KCNH2, including colony formation, CCK-8 proliferation, wound-healing, apoptosis assays, mitochondrial membrane potential measurement, and xenograft models, were performed to evaluate its biological effects.

**Results:**

Twenty-five intersecting targets were identified, and an eight-gene LASSO signature demonstrated favorable prognostic performance. Among these genes, KCNH2 showed consistent transcriptomic upregulation, proteomic validation, association with genomic instability, immune modulation patterns, and drug sensitivity correlations. Functional assays confirmed that KCNH2 silencing suppressed proliferation and migration-associated behavior while inducing mitochondria-dependent apoptosis in GBM cells. *In vivo*, KCNH2 knockdown significantly inhibited tumor growth.

**Conclusion:**

This integrative AI-driven and multi-omics strategy identifies KCNH2 as a biologically relevant and translationally informative candidate target potentially linked to the anti-glioma effects of Acorus tatarinowii. These findings highlight the value of combining traditional medicine resources with computational prioritization and experimental validation to uncover biologically actionable mechanisms in GBM.

## 1 Introduction

Glioblastoma (GBM) is the most aggressive primary malignant tumor of the central nervous system, characterized by rapid progression, extensive intratumoral heterogeneity, and profound therapeutic resistance (Rabah et al., 2023; Goenka et al., 2021). Despite current standard-of-care management—including maximal safe resection followed by radiotherapy and temozolomide—clinical outcomes remain poor, highlighting an urgent need for robust biomarkers that improve prognostic stratification and for actionable molecular targets that enable more effective therapeutic interventions (Sipos et al., 2025). Large-scale cancer genomics efforts have generated comprehensive molecular profiles of GBM; however, transforming these datasets into clinically meaningful biomarkers and drug targets remains a major translational challenge (Wang et al., 2021).

Recent advances in artificial intelligence (AI)–assisted modeling and integrative multi-omics analysis provide an opportunity to accelerate biomarker discovery and target prioritization. In particular, machine learning–based survival modeling can extract prognostically informative gene signatures from high-dimensional transcriptomic data, while multi-omics validation and downstream systems analyses can strengthen biological plausibility and translational relevance (Poursaeed et al., 2024). In parallel, network pharmacology has emerged as a useful framework to dissect the multi-component, multi-target characteristics of traditional Chinese medicine (TCM), enabling systematic prediction of herb–compound–target networks and potential therapeutic mechanisms (Zhai et al., 2025). Conceptually, integrating network pharmacology with AI-driven modeling offers a coherent strategy to link traditional therapeutic resources with modern computational oncology and to prioritize candidate targets for subsequent functional evaluation.

Acorus tatarinowii (Shi Chang Pu) is a classical TCM herb traditionally used for neurological and inflammatory conditions (Wang et al., 2023; Wen et al., 2023). Its bioactive constituents have been reported to exert neuroprotective, anti-inflammatory, and antioxidative effects, suggesting potential relevance to CNS disease biology (Balakrishnan et al., 2022). However, the molecular targets and pathways through which A. tatarinowii may be connected to GBM biology remain insufficiently defined, and pharmacology-informed target discovery has rarely been integrated with prognostic modeling and functional validation in GBM.

Among the candidate targets emerging from such integrative strategies, KCNH2 (also known as hERG) is of particular interest (Zheng and Song, 2023). KCNH2 encodes a voltage-gated potassium channel subunit that is classically implicated in cardiac electrophysiology, but growing evidence indicates that ion channels can regulate malignant phenotypes by shaping membrane potential, intracellular signaling, cell-cycle progression, and apoptotic susceptibility (Capatina et al., 2022; Yang et al., 2022). Aberrant KCNH2 expression has been reported across multiple cancer types and has been linked to proliferative and migratory phenotypes as well as unfavorable outcomes, suggesting that KCNH2 may represent a biologically relevant node rather than a passive marker (Zúñiga et al., 2022). Nevertheless, in GBM, the relationships between KCNH2 expression and clinically relevant phenotypes—including genomic instability, immune microenvironment features, pharmacogenomic vulnerabilities, and apoptosis regulation—remain incompletely characterized.

In this study, we developed an integrated workflow combining network pharmacology–based target prioritization, AI-assisted prognostic modeling (LASSO Cox regression), and multi-layer validation to identify GBM-relevant candidates and to select KCNH2 for functional interrogation. Using TCGA/GTEx transcriptomic resources and CPTAC proteomics, we evaluated expression and prognostic relevance of model genes and selected KCNH2 for further investigation. We then characterized copy-number alteration patterns, mutation landscape, immune microenvironment associations, and pharmacogenomic drug sensitivity correlations to contextualize KCNH2 within GBM biology. Finally, using genetic perturbation *in vitro* and *in vivo*, we show that KCNH2 suppression inhibits proliferation and migration-associated behavior and promotes mitochondria-dependent apoptosis, accompanied by reduced xenograft growth. Collectively, these results support KCNH2 as a prognostic biomarker and a therapeutically relevant candidate vulnerability in GBM, and they illustrate how integrating traditional medicine resources with AI-driven multi-omics can prioritize targets for downstream validation.

## 2 Methods

### 2.1 Data sources and preprocessing

GBM RNA-seq expression profiles and matched clinical survival information were obtained from TCGA (Cancer Genome Atlas Research Network, 2008). Normal brain tissue expression data were obtained from GTEx, and TCGA adjacent normal samples were included when available (The GTEx Consortium, 2020). To improve comparability across cohorts, UCSC Xena Toil-processed datasets were used for tumor–normal expression comparisons (Goldman et al., 2020; Vivian et al., 2017). Samples with missing overall survival time or survival status were excluded from survival analyses. Expression matrices were standardized by gene symbol harmonization, removal of duplicated genes (retaining the highest-expression entry where necessary), and log2 transformation when required by downstream analyses (Bateman et al., 2021; Tweedie et al., 2021). Clinical variables were checked for completeness and consistency, and survival time was unified to a consistent unit prior to modeling.

### 2.2 Network pharmacology-based compound screening and target prediction

Chemical constituents of Acorus tatarinowii (Shi Chang Pu) were retrieved from the Traditional Chinese Medicine Systems Pharmacology Database and Analysis Platform (TCMSP) (Ru et al., 2014). Candidate bioactive compounds were screened using oral bioavailability (OB) ≥ 30% and drug-likeness (DL) ≥ 0.18 (Wang et al., 2020). For compounds supported by published pharmacological evidence but not meeting these thresholds, manual inclusion was allowed after literature review to avoid missing well-studied constituents. Putative targets of selected compounds were collected from TCMSP, standardized to *Homo sapiens* entries using UniProt, converted to official gene symbols according to UniProt/HGNC conventions, and deduplicated to generate the final compound-related target list (Bateman et al., 2021).

### 2.3 Glioma-related gene collection, intersection targets, and preliminary prognostic filtering

Glioma/GBM-related genes were collected from multiple public resources, including GeneCards, OMIM, and the Therapeutic Target Database (TTD), using “glioma” and/or “glioblastoma” as keywords, followed by merging and deduplication to construct a comprehensive disease-associated gene set (Stelzer et al., 2016; Hamosh et al., 2021; Zhou et al., 2022). Intersection analysis between compound-related targets and disease-associated genes was performed to define candidate targets potentially relevant to GBM biology and to the putative anti-glioma activity of Acorus tatarinowii. The prognostic relevance of intersection genes was further evaluated by Cox regression in the TCGA-GBM cohort to provide an initial prioritization for subsequent prognostic modeling and downstream analyses.

### 2.4 Prognostic modeling: feature selection, risk score construction, and performance evaluation

To reduce multicollinearity and avoid overfitting, we performed LASSO Cox regression using the glmnet package with cross-validation to determine the optimal penalty parameter (λ), and genes with non-zero coefficients were retained as model features (Simon et al., 2011; Li et al., 2022). We then constructed a multivariable Cox regression model using the survival package, and stepwise refinement (step) was used when needed to obtain an optimal and parsimonious model. A risk score was calculated for each patient as the weighted sum of expression values and corresponding coefficients, and patients were dichotomized into high- and low-risk groups (median cutoff unless otherwise specified). Kaplan–Meier survival analysis with log-rank testing was used to compare survival between groups, and hazard ratios with 95% confidence intervals were estimated using Cox regression. Time-dependent ROC curves and AUC values at predefined time points were calculated using survivalROC (or equivalent implementation) to evaluate discriminative performance (Heagerty et al., 2000).

### 2.5 Expression and multi-omics validation

Tumor–normal expression differences for model genes were evaluated using appropriate tests based on data distribution (Wilcoxon rank-sum test or Student’s t-test) (The GTEx Consortium, 2020). For protein-level validation, CPTAC-based proteomics data were analyzed after removal of missing values, and tumor–normal protein expression differences were visualized using boxplots and assessed using non-parametric testing (Wang et al., 2021).

### 2.6 Functional enrichment analysis

GO and KEGG enrichment analyses were performed using clusterProfiler to explore the functional context of model genes (Yu et al., 2012). Enrichment significance was assessed by hypergeometric testing, followed by multiple-testing correction; adjusted P values below the predefined threshold were considered significant.

### 2.7 Genomic alteration analyses: CNV, mutation landscape, and CNV–expression association

Gene-level copy number alterations were obtained from TCGA resources generated by GISTIC2, and CNV states were categorized as deep deletion, shallow deletion, diploid, gain, or amplification (Mermel et al., 2011). Expression differences across CNV categories were assessed using Kruskal–Wallis testing. Somatic mutation data in MAF format were summarized using maftools, and variant classification, variant type, and SNV class distributions were visualized to characterize the mutation landscape of the gene(s) of interest (Mayakonda et al., 2018).

### 2.8 Immune microenvironment analyses

Immune infiltration estimates were collected from TIMER2.0 using multiple deconvolution algorithms (Li et al., 2020). Spearman correlation was used to evaluate associations between KCNH2 expression and inferred immune cell infiltration levels across algorithms, and results were summarized using heatmaps to compare algorithm consistency and immune contexture patterns.

### 2.9 Pharmacogenomic drug sensitivity association analysis

Drug response data were obtained from GDSC1/2 (IC50) and CTRP/PRISM (AUC) (Yang et al., 2013; Iorio et al., 2016; Basu et al., 2013; Corsello et al., 2020). Spearman correlation was used to assess associations between KCNH2 expression and drug response across cell lines. Negative correlations were interpreted as increased sensitivity with higher gene expression, whereas positive correlations suggested relative resistance. Compounds were ranked by statistical significance and effect size, and top candidates were visualized using lollipop-style plots.

### 2.10 Cell lines and cell culture

The human glioblastoma cell lines U87MG (RRID: CVCL_0022) and LN229 (RRID: CVCL_0393), as well as the human embryonic kidney cell line HEK293T (RRID: CVCL_0063), were obtained from the American Type Culture Collection (ATCC, Manassas, VA, USA). Normal human astrocytes NHA (RRID: CVCL_5792) were purchased from Lonza (Cat. No. CC-2565, Basel, Switzerland). U87MG and LN229 cells were maintained in Dulbecco’s Modified Eagle Medium (DMEM, Gibco, Cat. No. 11965092) supplemented with 10% fetal bovine serum (FBS, Gibco, Cat. No. 10099141C) and 1% penicillin-streptomycin solution (Gibco, Cat. No. 15140122). HEK293T cells were cultured under identical conditions. NHA cells were grown in Astrocyte Growth Medium (Lonza, Cat. No. CC-3186) according to the supplier’s instructions. All cells were incubated at 37 °C in a humidified atmosphere containing 5% CO_2_. Cells were passaged every 2–3 days and routinely tested for *mycoplasma* contamination prior to experimental use.

### 2.11 Generation of stable KCNH2-knockdown glioblastoma cell lines

Short hairpin RNA (shRNA) sequences targeting human KCNH2 and a non-targeting control sequence were designed and synthesized by GeneChem (Shanghai, China). The shRNA oligonucleotides were cloned into the lentiviral vector pLKO.1-puro (Addgene, Cat. No. 8453). For lentiviral packaging, HEK293T cells (RRID: CVCL_0063) were seeded in 10-cm dishes and co-transfected with the shRNA plasmid, psPAX2 packaging plasmid (Addgene, Cat. No. 12260), and pMD2.G envelope plasmid (Addgene, Cat. No. 12259) at a ratio of 4:3:1 using Lipofectamine™ 3000 Transfection Reagent (Invitrogen, Cat. No. L3000015). After 6 h, the medium was replaced with fresh complete DMEM. Viral supernatants were collected at 48 and 72 h post-transfection, centrifuged to remove cell debris, and filtered through a 0.45 μm membrane (Millipore, Cat. No. SLHV033RS). U87MG and LN229 cells were infected with the viral supernatant in the presence of 8 μg/mL polybrene (Sigma-Aldrich, Cat. No. TR-1003-G). After 24 h, the medium was replaced with fresh culture medium. Stable transfectants were selected with puromycin (Sigma-Aldrich, Cat. No. P8833) at a concentration of 1–2 μg/mL for 5–7 days. Knockdown efficiency was verified by quantitative real-time PCR prior to subsequent experiments.

### 2.12 Quantitative real-time PCR

Total RNA was isolated from cultured cells using TRIzol™ Reagent (Invitrogen, Cat. No. 15596026) following the manufacturer’s instructions. RNA concentration and purity were determined using a NanoDrop 2000 spectrophotometer (Thermo Scientific). First-strand cDNA was synthesized from 1 μg of total RNA using the PrimeScript™ RT Reagent Kit with gDNA Eraser (Takara, Cat. No. RR047A) to remove genomic DNA contamination. Quantitative real-time PCR was performed using TB Green® Premix Ex Taq™ II (Takara, Cat. No. RR820A) on a QuantStudio 5 Real-Time PCR System (Applied Biosystems). The amplification conditions were as follows: initial denaturation at 95 °C for 30 s, followed by 40 cycles of 95 °C for 5 s and 60 °C for 30 s. Melt curve analysis was conducted to confirm amplification specificity. Relative gene expression levels were calculated using the 2^−ΔΔCt^ method, with GAPDH as the internal control. Each experiment was performed with at least three independent biological replicates. The primer sequences used in this study were as follows:

GAPDH-F: GAC​AGT​CAG​CCG​CAT​CTT​CT
GAPDH-R: GCG​CCC​AAT​ACG​ACC​AAA​TC
KCNH2-F: TGG​ACA​CCA​TCA​TCC​GCA​AG
KCNH2-R: ATG​GCT​GTC​ACT​TCG​TCC​AG
BAX-F: TTC​ATC​CAG​GAT​CGA​GCA​GG
BAX-R: TGT​CCA​GCC​CAT​GAT​GGT​TC
BCL2-F: AAA​AAT​ACA​ACA​TCA​CAG​AGG​AAG​T
BCL2-R: TCC​CGG​TTA​TCG​TAC​CCT​GT
BCL2L1-F: CGG​ATT​TGA​ATC​TCT​TTC​TCT​CCC
BCL2L1-R: CGA​CCC​CAG​TTT​ACC​CCA​TC

### 2.13 Cell colony formation

For colony formation analysis, U87MG and LN229 stable cell lines were seeded into 6-well plates at a density of 800 cells per well and cultured for 10–14 days until visible colonies formed. The culture medium was replaced every 3 days. Cells were washed with PBS (Gibco, Cat. No. 10010023), fixed with 4% paraformaldehyde (Solarbio, Cat. No. P1110) for 20 min at room temperature, and stained with 0.1% crystal violet solution (Sigma-Aldrich, Cat. No. C0775) for 15 min. Colonies were rinsed, air-dried, and counted under a light microscope, with colonies containing >50 cells considered valid. All experiments were performed in triplicate.

### 2.14 Cell viability assay (CCK-8)

Cell viability was assessed using a Cell Counting Kit-8 (CCK-8; Dojindo Laboratories, Cat. No. CK04, Kumamoto, Japan) according to the manufacturer’s protocol. Briefly, U87MG and LN229 stable cell lines were seeded into 96-well plates at a density of 2 × 10^3^ cells per well in 100 μL complete medium and allowed to attach overnight. Cell viability was measured at 0, 24, 48, 72, and 96 h post-seeding; the 0 h time point was defined as the measurement taken at the start of the assay. At each indicated time point, 10 μL of CCK-8 reagent was added directly to each well, followed by incubation at 37 °C for 1–2 h in the dark. Absorbance was recorded at 450 nm using a microplate reader (BioTek Synergy H1, Agilent). Background absorbance was corrected using blank wells containing medium and CCK-8 reagent without cells. OD values were normalized to the shNC group at each corresponding time point, and relative cell viability was calculated accordingly. Each experimental condition was performed in five technical replicates and repeated in three independent biological experiments.

### 2.15 Wound healing assay

Cell migration capacity was evaluated using a wound healing assay. U87MG and LN229 stable cell lines were seeded into 6-well plates at a density of 5 × 10^5^ cells per well and cultured in complete medium until reaching ∼90–100% confluence. A linear wound was generated across the monolayer using a sterile 200 μL pipette tip. Detached cells were removed by washing twice with PBS, and the medium was replaced with serum-reduced DMEM containing 1% FBS to minimize proliferation-related effects on wound closure. Images were captured at 0 and 48 h using an inverted microscope (Olympus CKX53). Wound width was measured in multiple randomly selected fields per well using ImageJ (NIH), and the migration rate (wound closure) was calculated as: Wound closure (%) = [(initial wound width − wound width at indicated time)/initial wound width] × 100%. All experiments were performed in triplicate with at least three independent biological replicates.

### 2.16 siRNA transfection

Small interfering RNAs (siRNAs) targeting human KCNH2 (siKCNH2-1, siKCNH2-2, and siKCNH2-3) and a non-targeting negative control (siNC) were synthesized by GenePharma (Shanghai, China). Cells were seeded into 6-well plates and transfected at ∼50–60% confluence using Lipofectamine™ RNAiMAX (Invitrogen, Cat. No. 13778150). siRNA and RNAiMAX were diluted separately in Opti-MEM™ (Gibco, Cat. No. 31985062), mixed, and incubated for 10–15 min at room temperature before addition to cells (final siRNA concentration, 50 nM). After 6 h, the medium was replaced with fresh complete medium. Cells were harvested 48 h post-transfection for subsequent assays.

### 2.17 Annexin V/PI apoptosis assay

Apoptosis was evaluated using an Annexin V-APC/Propidium Iodide (PI) Apoptosis Detection Kit (BD Biosciences, Cat. No. 550474). U87MG and LN229 cells were collected 48 h after transfection, and both detached and adherent cells were harvested. Cells were washed twice with ice-cold PBS and resuspended in 1× binding buffer at 1 × 10^6^ cells/mL. For staining, 100 μL of cell suspension was incubated with 5 μL Annexin V-APC and 5 μL PI for 15 min at room temperature in the dark, followed by addition of 400 μL binding buffer. Samples were analyzed using a BD FACSCanto II flow cytometer. A minimum of 10,000 events per sample were acquired. Data were analyzed using FlowJo (Version 10.8). Early apoptosis was defined as Annexin V-APC positive/PI negative, and late apoptosis as Annexin V-APC positive/PI positive. Total apoptosis was calculated as the sum of early and late apoptotic populations. All experiments were conducted with at least three independent biological replicates.

### 2.18 Measurement of mitochondrial membrane potential

Mitochondrial membrane potential (ΔΨm) was assessed using tetramethylrhodamine methyl ester (TMRM; Abcam, Cat. No. ab113852). U87MG and LN229 cells were collected 48 h after siRNA transfection and incubated with 100 nM TMRM in serum-free DMEM for 25 min at 37 °C in the dark. Cells were washed once with pre-warmed PBS and analyzed immediately by flow cytometry (BD FACSCanto II). TMRM was excited at 488 nm and emission collected in the PE channel (∼585 nm). At least 10,000 events were acquired per sample. Mean fluorescence intensity (MFI) was quantified using FlowJo (Version 10.8) and normalized to the control group. All experiments were performed in three independent biological replicates.

### 2.19 Cleaved caspase-3 ELISA assay

Cleaved caspase-3 was quantified using the PathScan® Cleaved Caspase-3 (Asp175) Sandwich ELISA Kit (Cell Signaling Technology, Cat. No. 7190). U87MG and LN229 cells were harvested 48 h after siRNA transfection and lysed using the kit lysis buffer on ice for 30 min. Lysates were centrifuged at 12,000 × g for 10 min at 4 °C and supernatants collected. Total protein concentration was determined using a BCA Protein Assay Kit (Thermo Scientific, Cat. No. 23227), and equal amounts of protein were used for ELISA. Absorbance was measured at 450 nm using a microplate reader (BioTek Synergy H1, Agilent). Cleaved caspase-3 levels were expressed as relative values normalized to the control group. All measurements were performed in triplicate and repeated in at least three independent experiments.

### 2.20 Subcutaneous xenograft tumor model

All animal experiments were approved by the Institutional Animal Care and Use Committee (IACUC) and performed in accordance with institutional guidelines. Four- to six-week-old male BALB/c nude mice were purchased from GemPharmatech Co., Ltd. (Nanjing, China) and maintained under SPF conditions with controlled temperature/humidity and a 12 h light/dark cycle. Stable KCNH2-knockdown and control U87MG cells were harvested during logarithmic growth, washed with sterile PBS, and resuspended in PBS at 5 × 10^6^ cells per 100 μL. Each mouse received a subcutaneous injection into the right flank. Tumor growth was monitored every 3 days using digital calipers, and tumor volume was calculated as: Volume = (length × width^2^)/2. At the endpoint, mice were euthanized, tumors were excised, photographed, and weighed.

### 2.21 Statistical analysis

All statistical analyses were performed using R version 4.5.1 and GraphPad Prism when applicable. Data are presented as mean ± SD unless otherwise stated. Two-group comparisons were conducted using Student’s t-test or Wilcoxon rank-sum test based on distributional assumptions. Multi-group comparisons were performed using one-way ANOVA with appropriate *post hoc* testing or Kruskal–Wallis test. Survival differences were assessed by Kaplan-Meier analysis with log-rank testing, and Cox regression was used to estimate hazard ratios with 95% confidence intervals. Correlations were evaluated using Spearman’s method. All tests were two-sided, and P < 0.05 was considered statistically significant.

## 3 Results

### 3.1 Identification of intersecting targets between Acorus tatarinowii and glioma

**FIGURE 1:**
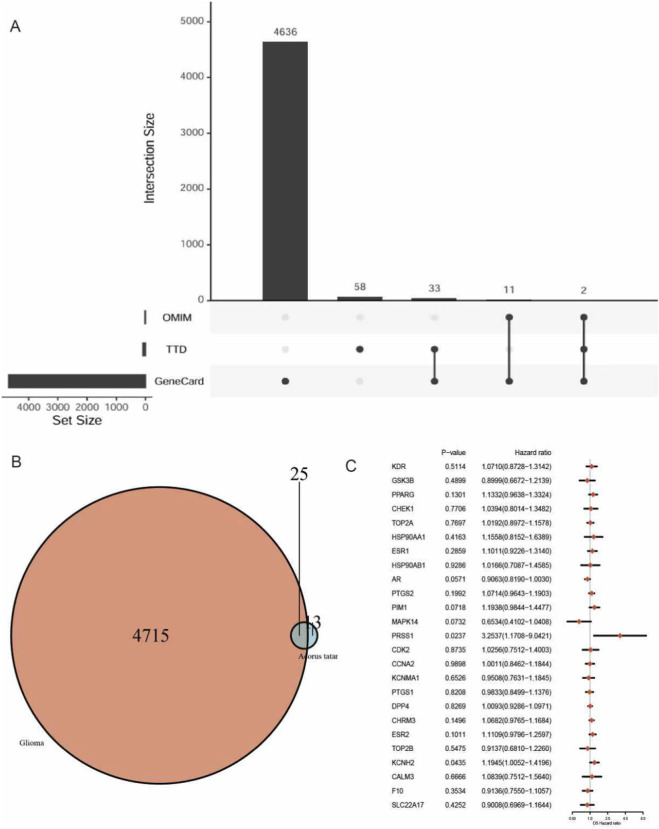
Identification of candidate targets of Acorus tatarinowii potentially relevant to glioma. **(A)** UpSet plot showing glioma-related genes retrieved from GeneCards, OMIM, and TTD and their overlaps across databases. **(B)** Venn diagram displaying the intersection between predicted A. tatarinowii targets and glioma-related genes; 25 overlapping genes were identified as candidate targets. **(C)** Forest plot of univariate Cox regression for the 25 candidate genes in the TCGA glioma cohort. Hazard ratios (HRs) with 95% confidence intervals (CIs) and P-values are shown. PRSS1 and KCNH2 were significantly associated with overall survival (P < 0.05).

Using TCMSP and the screening thresholds of OB ≥ 30% and DL ≥ 0.18, four candidate bioactive compounds of A. tatarinowii were retained for downstream analysis. Putative targets of these compounds were collected from TCMSP and standardized in UniProt (*H. sapiens*), yielding 38 compound-related targets after mapping to official gene symbols and removing duplicates. Glioma-related genes were compiled from GeneCards, OMIM, and TTD and merged into a unified gene set (Figure 1A). Intersection analysis between the A. tatarinowii target set and the glioma gene set identified 25 overlapping genes, which were considered candidate targets potentially mediating the effects of A. tatarinowii in glioma (Figure 1B). Univariate Cox regression was further performed to evaluate the prognostic relevance of these candidates in the TCGA-GBM cohort (Figure 1C).

### 3.2 Construction and functional characterization of the LASSO-based prognostic signature in GBM

**FIGURE 2:**
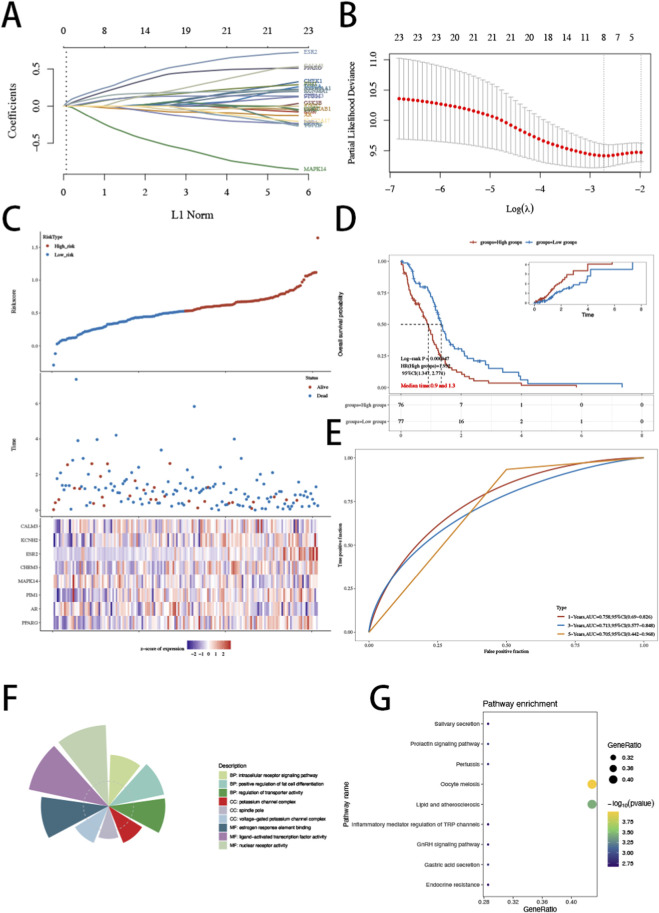
Construction and evaluation of a LASSO-derived prognostic signature and functional annotation of model genes. **(A)** LASSO coefficient profiles of candidate genes. Each curve represents a gene coefficient trajectory as the penalty increases. **(B)** Ten-fold cross-validation curve of partial likelihood deviance versus log(λ); the model at lambda.min = 0.0655 was selected. **(C)** Distribution of risk scores (top), survival status and follow-up time (middle), and expression heatmap of the eight model genes (bottom) across patients ranked by risk score. **(D)** Kaplan–Meier overall survival curves for high- and low-risk groups stratified by the median risk score, with the log-rank test and Cox regression results (log-rank P = 0.000447; HR = 1.952; 95% CI: 1.347–2.771). The inset shows cumulative hazard. **(E)** Time-dependent ROC curves for 1-, 3-, and 5-year survival prediction (AUC = 0.758, 0.713, and 0.705, respectively). **(F)** GO enrichment summary (BP/CC/MF) of the eight model genes. **(G)** KEGG pathway enrichment of the eight model genes; dot size indicates gene ratio and color indicates −log10 (P value).

To construct a prognostic model for GBM, we performed LASSO Cox regression with ten-fold cross-validation. As the penalty parameter λ increased, gene coefficients progressively shrank toward zero (Figure 2A). The optimal model was determined at lambda.min = 0.0655 based on the minimum cross-validated partial likelihood deviance (Figure 2B). Eight genes were retained to establish the risk model: PPARG, AR, PIM1, MAPK14, CHRM3, ESR2, KCNH2, and CALM3. A risk score was calculated for each patient using the corresponding regression coefficients. Risk score distribution, survival status, and gene expression patterns demonstrated that patients in the high-risk group exhibited increased mortality and shorter survival time (Figure 2C). Kaplan-Meier analysis revealed significantly worse overall survival in the high-risk group (log-rank P = 0.000447) (Figure 2D). Cox regression confirmed the prognostic value of the risk score (HR = 1.952, 95% CI: 1.347–2.771). Time-dependent ROC analysis demonstrated favorable predictive performance, with AUC values of 0.758, 0.713, and 0.705 at 1, 3, and 5 years, respectively (Figure 2E). Functional enrichment analysis suggested that the eight model genes were significantly involved in interleukin receptor signaling, regulation of transporter activity, and potassium channel complex–related functions (Figure 2F). KEGG analysis revealed enrichment in prolactin signaling, GnRH signaling, inflammatory mediator regulation of TRP channels, and endocrine resistance pathways (Figure 2G), indicating potential involvement in immune modulation, hormonal signaling, and ion channel–associated biological processes in GBM.

### 3.3 Expression and prognostic significance of model genes in GBM

**FIGURE 3:**
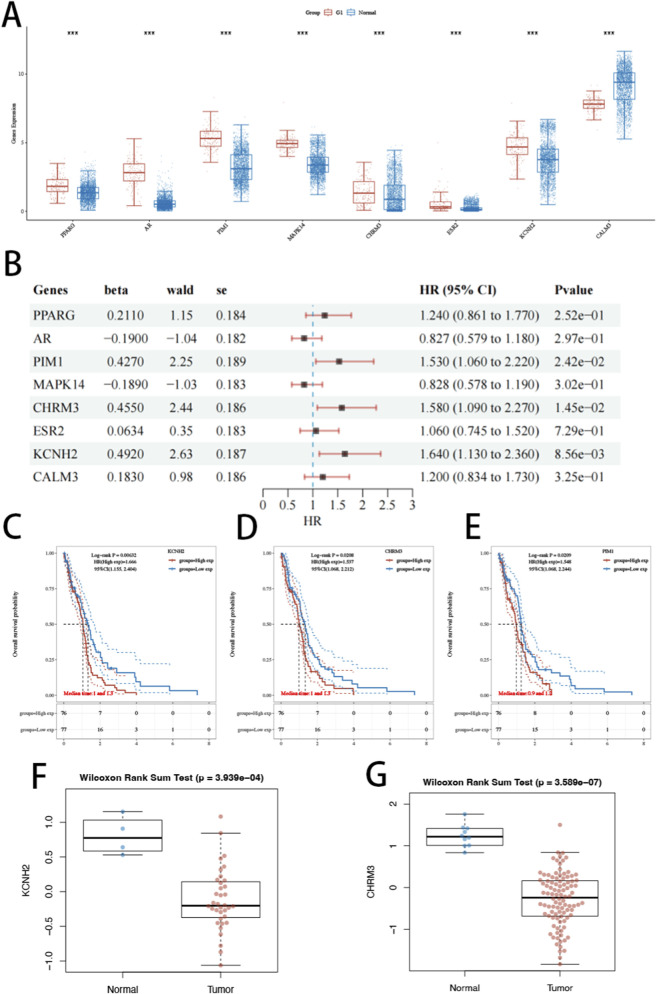
Expression patterns and prognostic significance of LASSO-selected genes in GBM. **(A)** Differential expression analysis of eight model genes (PPARG, AR, PIM1, MAPK14, CHRM3, ESR2, KCNH2, and CALM3) between normal brain tissues and GBM samples. **(B)** Forest plot showing hazard ratios (HRs) and 95% confidence intervals derived from univariate Cox regression analysis. **(C–E)** Kaplan–Meier survival curves of prognostically significant genes in GBM. **(F,G)** Protein expression levels of candidate genes in GBM and normal tissues based on CPTAC data. Statistical comparisons were performed using the Wilcoxon rank-sum test.

We first systematically examined the transcriptional expression patterns of the eight LASSO-selected genes (PPARG, AR, PIM1, MAPK14, CHRM3, ESR2, KCNH2, and CALM3) in GBM and normal brain tissues (Figure 3A). Compared with normal controls, PPARG, AR, PIM1, MAPK14, CHRM3, ESR2, and KCNH2 were significantly upregulated in GBM, whereas CALM3 exhibited reduced expression in tumor tissues. We next evaluated the prognostic significance of these eight genes using Cox regression and Kaplan–Meier survival analyses (Figures 3B–E). Among them, only KCNH2 (P = 8.56e−03, HR = 1.640, 95% CI: 1.130–2.360), CHRM3 (P = 1.45e−02, HR = 1.580, 95% CI: 1.090–2.270), and PIM1 (P = 2.42e−02, HR = 1.530, 95% CI: 1.060–2.220) showed significant associations with overall survival, with elevated expression corresponding to poorer clinical outcomes. Kaplan–Meier curves consistently demonstrated reduced survival probability in the high-expression groups of these three genes. To further validate their translational relevance at the protein level, we analyzed CPTAC proteomic data and observed that KCNH2 and CHRM3 displayed significantly higher protein expression in GBM tumor tissues compared with normal tissues (Figures 3F,G). In contrast, PIM1 did not show consistent protein-level validation. Taken together, these findings indicate that KCNH2, CHRM3, and PIM1 are prognostically relevant genes in TCGA-GBM, and among them, KCNH2 shows the most consistent support across transcriptomic expression, survival association, and proteomic validation. Therefore, we selected KCNH2 for subsequent in-depth genomic, immune, and experimental investigations.

### 3.4 Copy-number alteration, genomic instability, and mutation landscape of KCNH2 in GBM

**FIGURE 4:**
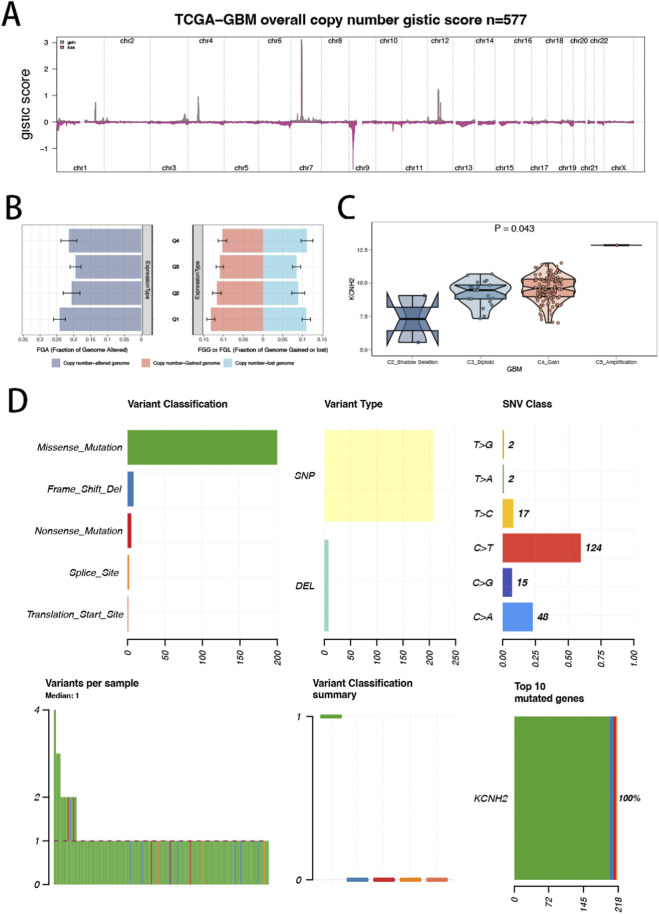
Copy-number alteration, genomic instability, and mutation landscape of KCNH2 in TCGA-GBM. **(A)** Genome-wide GISTIC2 copy-number profile in TCGA-GBM, showing recurrent chromosomal gains and losses across the cohort. **(B)** Comparison of genomic instability metrics across KCNH2 expression quartiles (Q1–Q4), including fraction genome altered (FGA) and fractions of genome gained (FGG) or lost (FGL). **(C)** KCNH2 expression across discrete GISTIC2 copy-number states (e.g., shallow deletion, diploid, gain, amplification). **(D)** Somatic mutation summary of KCNH2, including variant classification, variant type, SNV class distribution, and variants per sample.

Genome-wide GISTIC2 analysis revealed widespread copy-number alterations across the TCGA-GBM cohort (Figure 4A). Stratification by KCNH2 expression quartiles (Q1–Q4) demonstrated a stepwise increase in genomic instability metrics, including fraction of genome altered (FGA), fraction gained (FGG), and fraction lost (FGL), in higher KCNH2 expression groups (Figure 4B). This indicates that elevated KCNH2 expression is associated with increased chromosomal instability. Consistently, KCNH2 expression differed significantly across CNV states (P = 0.043) and tended to increase from deletion/diploid states toward gain/amplification states (Figure 4C), supporting a gene dosage effect. Mutation profiling showed that missense mutations represented the predominant variant type, with additional low-frequency events including frameshift deletion, nonsense, splice-site, and start-site mutations. Single nucleotide variants were primarily characterized by C > T substitutions (Figure 4D). These results suggest that KCNH2 is linked to CNA activity and exhibits a defined somatic mutation spectrum in GBM.

### 3.5 Association between KCNH2 expression and immune landscape in GBM

**FIGURE 5:**
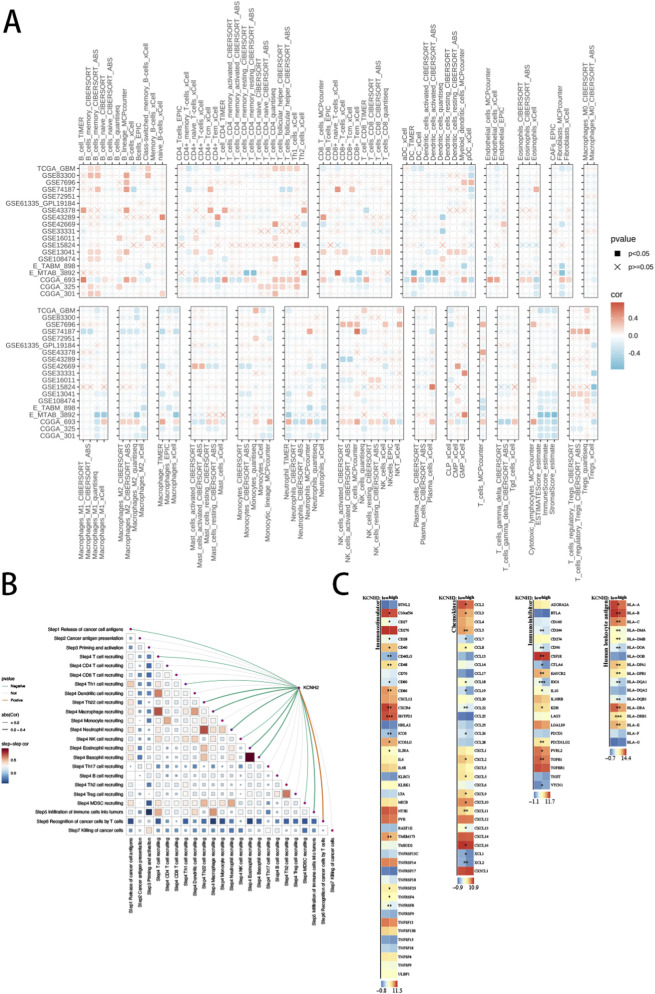
Immune landscape associated with KCNH2 expression in GBM. **(A)** Spearman correlation heatmap showing the association between KCNH2 expression and immune cell infiltration estimated by multiple algorithms in TCGA-GBM. Colored squares represent significant correlations, with red indicating positive and blue indicating negative correlations; non-significant correlations are marked. **(B)** Correlation network between KCNH2 expression and Tumor Immunophenotype (TIP) scores, as well as autocorrelation among TIP components. Line color and thickness represent correlation direction and magnitude. **(C)** Heatmap showing differential expression of immunostimulatory genes, immunoinhibitory genes, chemokines, and HLA molecules between high- and low-KCNH2 expression groups in GBM.

We first examined the correlation between KCNH2 expression and immune cell infiltration across multiple computational algorithms in TCGA-GBM (Figure 5A). Spearman correlation analysis revealed that KCNH2 expression was differentially associated with various immune cell types, with both positive and negative correlations observed depending on the algorithm used. Although correlation strength varied across methods, consistent associations were detected for several immune subsets, suggesting that KCNH2 expression is closely linked to the immune microenvironment of GBM. We next evaluated the relationship between KCNH2 expression and cancer immunity cycle activity using TIP scores (Figure 5B). KCNH2 expression showed significant correlations with multiple TIP components, suggesting an association with specific stages of the tumor–immune cycle in GBM. Correlation analysis among TIP scores further demonstrated coordinated immune activity patterns within GBM. Finally, we compared the expression of immunostimulatory genes, immunoinhibitory genes, chemokines, and HLA molecules between high- and low-KCNH2 expression groups (Figure 5C). The high-KCNH2 group exhibited a broadly altered immune-related gene expression profile, with multiple immune regulatory molecules showing significant differences between groups. Collectively, these findings indicate that KCNH2 expression is associated with immune-related features in GBM, although these correlations should be interpreted as exploratory rather than treatment-predictive.

### 3.6 Association between KCNH2 expression and drug sensitivity in GBM

**FIGURE 6:**
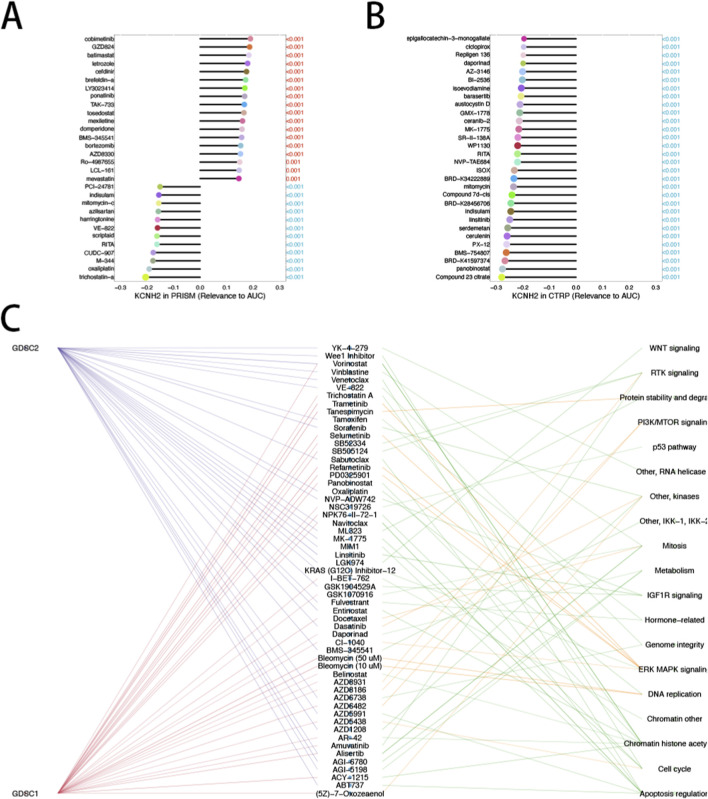
Drug sensitivity analysis associated with KCNH2 expression. **(A,B)** Spearman correlation between KCNH2 expression and drug response metrics across GDSC1, GDSC2, PRISM, and CTRP datasets. The x-axis represents correlation coefficients, and the y-axis shows the top 30 most significant drugs. Bar length reflects correlation magnitude. **(C)** Drug class enrichment analysis of significantly correlated compounds. Bubble size represents gene ratio, and color indicates statistical significance.

Spearman correlation analysis across GDSC1, GDSC2, PRISM, and CTRP datasets identified multiple compounds whose IC50 or AUC values were significantly correlated with KCNH2 expression (Figures 6A,B). Both positive and negative correlations were observed, indicating that KCNH2 expression may confer context-dependent drug sensitivity or resistance. Drug class enrichment analysis of significantly associated compounds revealed enrichment in agents targeting cell cycle regulation, DNA damage response pathways, and kinase signaling networks (Figure 6C). These findings suggest that KCNH2 expression may be linked to pharmacogenomic response patterns and could help prioritize candidate vulnerabilities for further validation in GBM.

### 3.7 KCNH2 is upregulated in glioblastoma cells and efficiently silenced by shRNA

**FIGURE 7:**
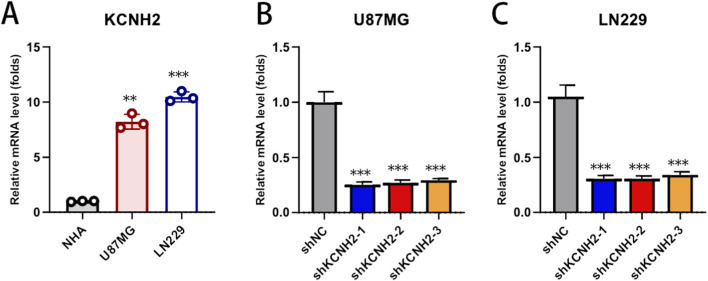
Differential expression of KCNH2 and validation of stable knockdown efficiency in glioblastoma cells. **(A)** Relative mRNA expression of KCNH2 in NHA, U87MG, and LN229 cells measured by quantitative PCR. **(B,C)** Relative KCNH2 mRNA levels in U87MG and LN229 cells transduced with shNC or three independent shRNAs targeting KCNH2. Data are presented as mean ± SD from three independent experiments.

KCNH2 mRNA expression was first compared between normal human astrocytes (NHA) and glioblastoma cell lines (U87MG and LN229). KCNH2 levels were markedly elevated in both U87MG and LN229 cells relative to NHA, indicating aberrant upregulation of KCNH2 in glioblastoma cells (Figure 7A). To investigate the functional relevance of KCNH2, stable knockdown models were generated using three independent shRNAs in U87MG and LN229 cells. All three shRNAs significantly reduced KCNH2 mRNA expression compared with the shNC group in both cell lines, confirming efficient and reproducible silencing of KCNH2 (Figures 7B,C). These validated knockdown models were subsequently used for downstream functional experiments.

### 3.8 KCNH2 depletion suppresses clonogenic growth, proliferation, and migration of glioblastoma cells

**FIGURE 8:**
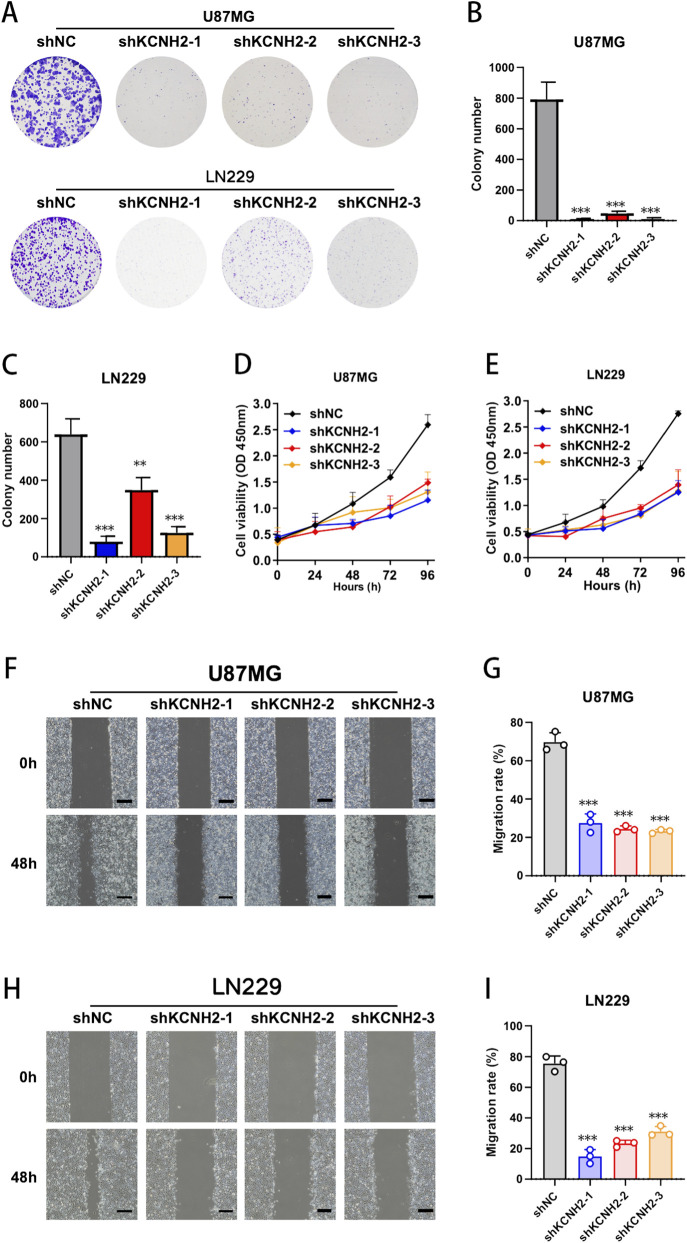
KCNH2 knockdown impairs clonogenicity, proliferation, and migration in glioblastoma cells. **(A–C)** Representative colony formation images and quantitative analysis of colony numbers in U87MG and LN229 stable cell lines transduced with shNC or three independent shRNAs targeting KCNH2. **(D,E)** CCK-8 growth curves of U87MG and LN229 cells following KCNH2 silencing, showing reduced cell viability over time. **(F–I)** Representative wound-healing images at 0 h and 48 h and quantitative analysis of migration rates in U87MG and LN229 cells. Data are presented as mean ± SD (n = 3).

Silencing of KCNH2 significantly impaired the clonogenic potential of glioblastoma cells. In U87MG cells, shKCNH2-1, shKCNH2-2, and shKCNH2-3 all led to a dramatic reduction in colony formation compared with shNC controls. Similar inhibitory effects were observed in LN229 cells, where KCNH2 knockdown markedly decreased both colony density and colony size. Quantitative analysis confirmed a robust decline in colony numbers across all three independent shRNA constructs in both cell lines, indicating that KCNH2 is essential for sustained clonogenic survival (Figures 8A–C). Consistent with these findings, CCK-8 assays demonstrated that KCNH2 depletion significantly attenuated cell proliferation over time. In both U87MG and LN229 cells, growth curves in the shKCNH2 groups displayed a noticeably flatter trajectory compared with shNC cells, particularly at later time points, suggesting impaired proliferative expansion following KCNH2 silencing (Figures 8D,E). We further evaluated migratory capacity using wound-healing assays. While control cells exhibited substantial wound closure within 48 h, KCNH2 knockdown cells retained a markedly wider wound gap in both U87MG and LN229 models. Quantitative assessment confirmed a significant reduction in wound closure rates in all shKCNH2 groups relative to shNC controls, supporting a migration-associated phenotype under serum-reduced conditions (Figures 8F–I). Taken together, these results establish that KCNH2 promotes clonogenic growth, proliferative capacity, and migratory behavior in glioblastoma cells.

### 3.9 KCNH2 silencing activates intrinsic mitochondrial apoptosis in glioblastoma cells

**FIGURE 9:**
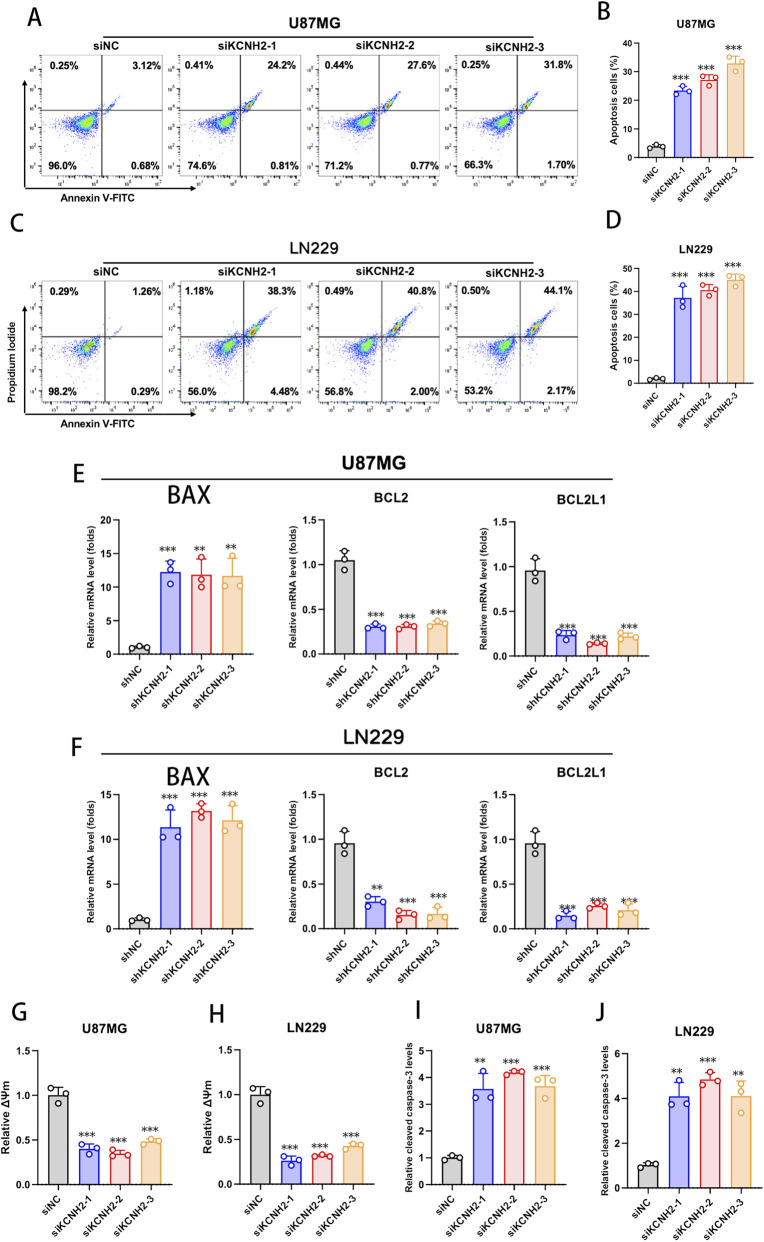
KCNH2 silencing triggers mitochondria-dependent apoptosis in glioblastoma cells. **(A,C)** Representative Annexin V-FITC/PI flow cytometry plots in U87MG **(A)** and LN229 **(C)** cells transfected with siNC or three independent siRNAs targeting KCNH2 (siKCNH2-1/2/3). **(B,D)** Quantification of total apoptotic cells (early + late apoptosis) in U87MG **(B)** and LN229 **(D)**. **(E,F)** Relative mRNA expression levels of mitochondrial apoptosis-related genes (BAX, BCL2, BCL2L1) in U87MG **(E)** and LN229 **(F)**; transcript levels were normalized to GAPDH. **(G,H)** Mitochondrial membrane potential (ΔΨm) assessed by TMRM staining in U87MG **(G)** and LN229 **(H)**; fluorescence intensity was normalized to the control group. **(I,J)** Cleaved caspase-3 levels measured by ELISA in U87MG **(I)** and LN229 **(J)**. Data are presented as mean ± SD (n = 3). Statistical significance is indicated in the figure.

To determine whether the growth-suppressive effect of KCNH2 depletion was mediated by apoptosis, we performed Annexin V/PI flow cytometric analysis. In both U87MG and LN229 cells, KCNH2 silencing markedly increased apoptotic populations, with substantial elevations in both early apoptotic (Annexin V^+^/PI^−^) and late apoptotic (Annexin V^+^/PI^+^) fractions compared with control cells. Quantitative analysis demonstrated significantly higher total apoptosis rates across all three independent siKCNH2 groups (Figures 9A–D), indicating that KCNH2 depletion promotes apoptotic cell death. To explore the underlying mechanism, we next examined key regulators of mitochondrial apoptosis. KCNH2 knockdown significantly upregulated the pro-apoptotic gene BAX, while simultaneously downregulating the anti-apoptotic genes BCL2 and BCL2L1 in both glioblastoma cell lines (Figures 9E,F). This shift in the BAX/BCL2 axis suggests activation of the intrinsic apoptotic pathway. Consistently, TMRM staining revealed a pronounced reduction in mitochondrial membrane potential (ΔΨm) following KCNH2 silencing (Figures 9G,H), indicating mitochondrial depolarization—an early hallmark of intrinsic apoptosis. Furthermore, ELISA analysis showed significantly increased levels of cleaved caspase-3 in KCNH2-depleted cells (Figures 9I,J), confirming activation of the downstream execution phase of apoptosis. Collectively, these findings demonstrate that KCNH2 knockdown induces mitochondria-dependent apoptosis characterized by mitochondrial membrane depolarization, dysregulation of BCL2 family members, and caspase-3 activation in glioblastoma cells.

### 3.10 KCNH2 knockdown suppresses glioblastoma growth *in vivo*

**FIGURE 10:**
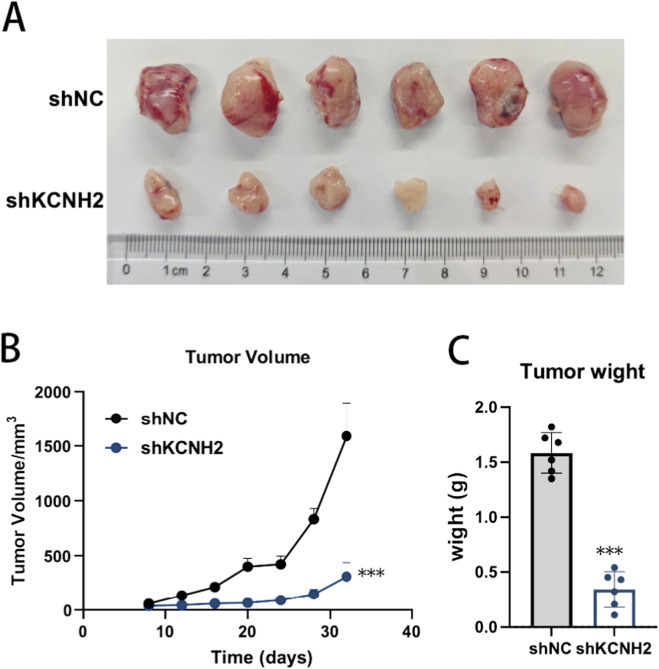
KCNH2 knockdown suppresses glioblastoma tumor growth *in vivo*. **(A)** Representative images of xenograft tumors excised from nude mice injected with shNC or shKCNH2 U87MG cells. **(B)** Tumor growth curves showing tumor volume progression over time. **(C)** Final tumor weights at the experimental endpoint. Data are presented as mean ± SD (n = 6). ***P < 0.001 versus shNC.

To further validate the tumor-suppressive effect of KCNH2 depletion *in vivo*, we established a subcutaneous xenograft model using U87MG cells stably transduced with shNC or shKCNH2. Tumors derived from shKCNH2 cells were visibly smaller than those formed by control cells at the experimental endpoint (Figure 10A). Longitudinal monitoring of tumor growth demonstrated a markedly reduced growth rate in the shKCNH2 group throughout the observation period (Figure 10B). While tumors in the control group exhibited rapid expansion, tumors in the KCNH2-depleted group showed significantly slower volume progression, with a pronounced divergence emerging during the later stages of tumor development. Consistent with these findings, final tumor weights were substantially lower in the shKCNH2 group compared with the shNC group at sacrifice (Figure 10C). Quantitative analysis confirmed that KCNH2 knockdown significantly reduced both tumor volume and tumor mass *in vivo*. Collectively, these results demonstrate that genetic suppression of KCNH2 inhibits glioblastoma growth in a xenograft model, supporting its functional relevance and motivating further evaluation of its therapeutic actionability.

## 4 Discussion

This study integrates network pharmacology–guided target prioritization with machine-learning prognostic modeling, multi-omics validation, and functional experiments to support KCNH2 as a GBM-relevant biomarker and a therapeutically informative candidate vulnerability (Morello et al., 2025). Starting from Acorus tatarinowii–derived candidate targets, we narrowed the candidate space to an 8-gene LASSO Cox signature and then further refined the biological focus by jointly considering tumor–normal expression patterns, survival relevance, and proteomic support. This “target-to-evidence-to-function” workflow reduces the risk of over-interpreting purely *in silico* hits and provides a coherent rationale for advancing KCNH2 to mechanistic validation in GBM.

A key observation is that KCNH2 shows concordant signals across multiple layers: elevated expression in GBM at the transcript level, prognostic relevance by survival analysis, and increased protein abundance in tumor tissues (Kim et al., 2024). This convergence is important because many ML-selected genes can be prognostic without being biologically central, or can be differentially expressed without translating to protein-level changes (Yanovich-Arad et al., 2021). By contrast, KCNH2 shows consistent support across transcriptomics, survival association, and proteomics, making it a more credible candidate node than targets supported by a single evidence layer.

Beyond association, our experiments support a functional role of KCNH2 in sustaining malignant phenotypes. Genetic suppression of KCNH2 decreases clonogenic survival, slows proliferative expansion, and impairs migratory capacity in glioblastoma cell models, indicating that KCNH2 contributes to both growth maintenance and invasive behavior (Shugg et al., 2021). Mechanistically, KCNH2 depletion increases apoptotic fractions and shifts mitochondrial apoptosis regulators toward a pro-death state (BAX up; BCL2/BCL2L1 down), accompanied by mitochondrial depolarization and increased cleaved caspase-3, supporting activation of mitochondria-dependent apoptosis (Benn et al., 2024). Together, these findings argue that KCNH2 supports GBM cell survival partly by restraining intrinsic, mitochondria-dependent apoptosis, providing a mechanistic bridge between prognostic association and tumor biology.

The genomic and microenvironment analyses add further context for why KCNH2-high tumors may behave more aggressively. The association between higher KCNH2 expression and greater copy-number alteration burden suggests that KCNH2 upregulation may track with a genomically unstable background, either as a downstream consequence of broad genomic remodeling or as part of an adaptive program that helps cells tolerate stress (Mazzoleni et al., 2024). In parallel, our immune analyses indicate that KCNH2 expression is associated with immune-contexture variation across algorithms and with differences in immunity-cycle activities and immune regulatory molecules (Avila Cobos et al., 2020). While these computational associations do not establish causality, they highlight that KCNH2 is not an isolated tumor-intrinsic marker and may sit at the interface of tumor state and immune tone—an angle that fits well with biomarker-oriented translational positioning.

From a therapeutic perspective, the pharmacogenomic correlations suggest that KCNH2 expression may stratify drug response patterns and could be explored as a hypothesis-generating companion biomarker for prioritizing candidates in GBM (Duan et al., 2024). This is particularly relevant for a target like KCNH2, which is a channel protein with existing pharmacology across multiple compound classes. At the same time, any attempt to therapeutically target KCNH2 must explicitly consider feasibility and safety: KCNH2 (hERG) is well-known for cardiac electrophysiology liability in systemic settings, so translational strategies would likely require careful dose control, brain-tumor selectivity, delivery innovations, or approaches that modulate downstream dependencies rather than directly blocking the channel in a non-selective manner.

Several limitations should be acknowledged. First, the prognostic model and downstream correlations are based on retrospective public datasets; additional external GBM cohorts and prospective evaluation would strengthen clinical generalizability. Second, algorithm-dependent variability in immune deconvolution emphasizes that immune associations should be interpreted as convergent trends rather than absolute quantities. Third, the experimental section uses knockdown-based approaches; complementary rescue experiments (re-expression) and orthogonal perturbations would further support specificity (Liu et al., 2021). Fourth, the *in vivo* validation uses a subcutaneous xenograft model, which does not fully recapitulate the brain microenvironment and blood–brain barrier constraints of GBM; orthotopic models would be an important next step for translational relevance. Despite these constraints, the multi-layer consistency and functional validation together support KCNH2 as a biomarker-linked candidate vulnerability in GBM and provide a framework for follow-up studies aimed at therapeutic actionability.

## 5 Conclusion

Our integrated network pharmacology and machine-learning framework identifies KCNH2 as a multi-omics–supported prognostic biomarker in GBM and demonstrates that genetic suppression of KCNH2 attenuates tumor-associated phenotypes through activation of mitochondria-dependent apoptosis. Collectively, these findings support KCNH2 as a biomarker-linked and biologically actionable candidate vulnerability in GBM and provide a rationale for further translational investigation.

## Data Availability

The original contributions presented in the study are included in the article/supplementary material, further inquiries can be directed to the corresponding author.
